# Pebble to the Metal: A Boulder Approach to Enrichment for *Danio rerio*

**DOI:** 10.1371/journal.pone.0298657

**Published:** 2024-05-07

**Authors:** Kyna A. Byrd, Jacob H. Theil, Jerome T. Geronimo, Jamie Ahloy-Dallaire, Michael F. Gutierrez, Emily I. Hui, Teagan K. Felt, Kendall M. Coden, Anna S. Ratuski, Stephen A. Felt, David K. Chu, Joseph P. Garner

**Affiliations:** 1 Department of Comparative Medicine, Stanford University, Stanford, California, United States of America; 2 Campus Veterinary Services, University of California, Davis, Davis, California, United States of America; 3 Department of Animal Science, Université Laval, Quebec, Quebec, Canada; 4 Department of Human and Organizational Development, Vanderbilt University, Nashville, Tennessee, United States of America; 5 Neuroscience Graduate Program, University of Michigan, Ann Arbor, Michigan, United States of America; 6 Department of Psychiatry and Behavioral Sciences, Stanford University, Stanford, California, United States of America; University Zürich, SWITZERLAND

## Abstract

Zebrafish are an established and widely used animal model, yet there is limited understanding of their welfare needs. Despite an increasing number of studies on zebrafish enrichment, in-tank environmental enrichment remains unpopular among researchers. This is due to perceived concerns over health/hygiene when it comes to introducing enrichment into the tank, although actual evidence for this is sparse. To accommodate this belief, regardless of veracity, we tested the potential benefits of enrichments presented outside the tank. Thus, we investigated the preferences and physiological stress of zebrafish with pictures of pebbles placed underneath the tank. We hypothesized that zebrafish would show a preference for enriched environments and have lower stress levels than barren housed zebrafish. In our first experiment, we housed zebrafish in a standard rack system and recorded their preference for visual access to a pebble picture, with two positive controls: visual access to conspecifics, and group housing. Using a crossover repeated-measures factorial design, we tested if the preference for visual access to pebbles was as strong as the preference for social contact. Zebrafish showed a strong preference for visual access to pebbles, equivalent to that for conspecifics. Then, in a second experiment, tank water cortisol was measured to assess chronic stress levels of zebrafish housed with or without a pebble picture under their tank, with group housing as a positive control. Cortisol levels were significantly reduced in zebrafish housed with pebble pictures, as were cortisol levels in group housed zebrafish. In fact, single housed zebrafish with pebble pictures showed the same cortisol levels as group housed zebrafish without pebble pictures. Thus, the use of an under-tank pebble picture was as beneficial as being group housed, effectively compensating for the stress of single housing. Pebble picture enrichment had an additive effect with group housing, where group housed zebrafish with pebble pictures had the lowest cortisol levels of any treatment group.

## Introduction

A rapidly growing literature clearly establishes that an animal’s overall well-being impacts not only the reproducibility, but ultimately the translatability of experiments [1]. Fish are not an exception to this, despite the dubious argument that their phylogenetic placement somehow justifies less ethical worth or protection [2]. In the United Kingdom [3] and Canada [4], where numbers of fish in research are tracked (unlike in the United States), fish are often the second most used vertebrate laboratory animal model behind mice. Of fish in general, zebrafish (*Danio rerio*) make up the vast majority used in research. Juxtaposed to their wild environments, standard laboratory housing conditions maintain zebrafish in barren environments. In the wild, zebrafish habitats are typically vegetated, slow-moving streams and ponds of Central Asia, with silt or gravel substrates [5]. There is a growing body of literature suggesting that zebrafish benefit from environmental enrichment [6]. The definition of environmental enrichment is widely inconsistent in literature [7]. However for the purposes of this paper, we are using the definition described in [8] where enrichment is both biologically relevant to the species it is being provided to, as well as beneficial to the animals’ overall well-being. In contrast to commonly used species in research (*e*.*g*., rodents) [9], enrichment is not widely or consistently implemented with zebrafish. Recent international survey work documents that less than half of zebrafish researchers are using in-tank enrichment [10]. The reason for their inaction is elucidated in the survey–researchers hesitate to use enrichment with zebrafish due to three perceived concerns: hygiene, health, and husbandry (although actual evidence that supports these concerns is sparse).

One way to sidestep these concerns is to use pictures of substrate under zebrafish tanks. Group housed zebrafish prefer tanks enriched by substrate (including pictures of gravel under the tank) and/or in-tank plants over barren tanks [11]. Zebrafish show a preference for compartments that had a gravel picture over a compartment that had white paper, and group housed zebrafish provided with a gravel picture show more positive behavioral responses, reflecting a more relaxed state of being [12]. Similar results are found for singly housed zebrafish, that show a preference for a marble picture under their tank [13]. While promising, these important preference papers have several limitations. These papers, as well as other preference test studies in this field, use barriers in the tank to measure preference and/or larger non-standard tanks, which may change zebrafish’s usual behavior and stress levels. Therefore, we cannot conclude that these preference results are relevant to zebrafish housed in standard, barrier-free housing conditions. Additionally, measures of behavior (*e*.*g*., preference tests) alone are not as meaningful without concurrent physiological measures (*e*.*g*., cortisol levels) to validate the results [14]. Specifically, a preference test alone may only show a zebrafish choosing between the lesser of two evils, where the preferred item is not actually providing the animal with any significant welfare benefit. Solutions to this problem include ‘calibrating’ the strength of a preference against a positive control (*i*.*e*., a known valued resource). To our knowledge, the work reported below is the first study that seeks to assess the benefits of under-tank pictures for zebrafish housed in barrier-free, rack-style aquatic housing systems, to calibrate that preference with a positive control, and to integrate preference with physiological measures. We hypothesized that if under-tank enrichment is beneficial in real-world conditions, preference should be maintained, and should be supported by other indicators of welfare such as physiology.

To test for and quantify potential benefits of under-tank enrichment for zebrafish, we first compared under-tank enrichment to the known preference for social housing. Zebrafish are social animals that form swimming groups with conspecifics [15] (*i*.*e*. “shoals”), both in the wild and in captivity [16]. Zebrafish show a preference for social housing and visual contact with conspecifics [6]. Approaching and shoaling with conspecifics is visually driven, and accordingly the species has excellent vision [17]. Therefore, we investigated both singly housed and group housed zebrafish’s behavioral responses to two types of visual environmental enrichment: 1) a pebble picture underneath their tanks and 2) visual access to conspecifics in neighboring tanks. In this crossover design, both group housing as well as visual access to conspecifics act as positive controls. Positive controls are essential best practice in animal welfare science, because they measure the value of a treatment (in this case, pebble pictures) against a known good (in this case, group housing and visual contact). We predicted that both singly and group housed zebrafish would prefer housing with a pebble picture, and prefer housing with visual access to conspecifics. We also predicted that visual access to conspecifics would be more important to the singly housed animals.

In a second experiment, we tested whether pebble pictures beneath the tank impacted stress physiology in singly housed and group housed zebrafish. To do so, we housed fish with or without pebble pictures under the entirety of their tank floor for one week. We measured cortisol levels in tank water, which is a well validated and non-invasive method for assessing chronic stress in zebrafish [18]. Again, the group housed zebrafish served as a positive control. We predicted that pebble-picture enriched tanks would lead to both singly and group housed zebrafish having lower chronic stress levels, as measured by tank water cortisol. In both experiments, comparing the effect of pebble enrichment to the effect of visual access or social housing puts any such effects into psychological and physiological context.

## Methods

### Ethics statement

All procedures were approved by Stanford’s Institutional Animal Care and Use Committee. Stanford is an AAALAC-accredited institution. Health checks were performed daily. All live-animal procedures were non-invasive and thus no analgesia or anesthesia was employed. On the final day of the study, zebrafish were euthanized via an ice bath of 4°C as described in the AVMA euthanasia guidelines.

We took additional steps to alleviate the welfare impact of this study in line with the 3Rs. In particular, the animals used were excess adults intended for our Sentinel program, and slated for euthanasia (*i*.*e*., a 3Rs Replacement) [19]. Furthermore, heterogenous population samples such as this, when properly analyzed, are more powerful, less prone to false positive results, more reproducible, and more likely to pertain to the general population (*i*.*e*., a 3Rs Reduction and Refinement) [1, 20–22]. We used non-invasive observational methods and tank-water cortisol sampling methods (*i*.*e*., a 3Rs Refinement). Finally, following best practice, we used complex split-plot and factorial designs and analyses which are much more powerful than t-tests [20]. Accordingly, sample size for both experiments was determined *a-priori* using Mead’s Rule, following best practice [20].

### Animals and housing

A total of 51 adult mixed-sex wildtype (AB strain) zebrafish, *Danio rerio*, were housed in 1.4-liter tanks and placed on a fixed recirculating aquatic system for the duration of this study. Prior to the experiment, the zebrafish were housed in barren, mixed-sex tanks at a density of approximately 5 zebrafish per liter. Experimental zebrafish (*N* = 40) were randomly assigned to 8 group housed tanks (4 zebrafish per tank) and 8 singly housed tanks. The remainder of zebrafish (*N* = 11) were housed singly (3 tanks) or in groups of 4 (2 tanks) and utilized as stimulus animals. Zebrafish were maintained on a 14:10 light:dark light cycle and fed twice daily with brine shrimp cultured in house. Water was maintained at conditions considered appropriate for the species (Temperature = 25–29°C, dissolved oxygen 6 mg/L, pH = 6.5–8.5, conductivity = 500–2000 μS, hardness = 175–200 mg/L, alkalinity = 50–150 mg/L, ammonia < 0.02 mg/L, nitrite < 0.05 mg/L, nitrate < 50 mg/L, monochloramine = 0.04–4.50 mg/L, copper = 1–210 μg/L). Tanks were arranged into groups of four so that the two middle tanks in each group had a group housed and singly housed neighbor. Tank order was varied to control for order effects, and each group had two singly housed and two group housed tanks. Both Experiment 1 and Experiment 2 were replicated across two racks. Clear rigid plastic covers with holes for feeding, water inflow, and condensation control were placed over the tops of tanks to prevent escape of zebrafish. Tables outlining the experimental design and treatment schedules for Experiments 1 and 2 are provided in the S1 File.

### Experiment 1: Zebrafish preference testing

In our first experiment, we measured zebrafish preference for an under-tank pebble pictures [23] using visual and/or physical access to conspecifics as a positive control. All tanks were filmed continuously throughout the study period by overhanging video cameras, recording two tanks per camera.

Preference tests were performed over a two-week period to assess predilection for substrate picture enrichment and visual access to conspecifics. To test preference for substrate pictures, pebble pictures were placed underneath tanks spanning half of the tank floor. The tanks were non-colored and transparent which allowed the pebble picture to be visible from inside the tank. Location of the pictures were alternated between the back and the front of the tanks to prevent location bias. To test for preference for conspecific visual access, white paper dividers were placed on the sides of tanks to allow for light to enter but exclude visual access to either the front half or back half of neighboring tanks. As with the pictures of pebbles, visual access was alternated between the back and the front of the tanks to prevent location bias. The side of the tank with pictures of pebbles or visual access to conspecifics is referred to as the “enriched side of the tank” throughout Experiment 1. Stimulus zebrafish were placed next to all end tanks so that all zebrafish had visual access to neighbors on both sides. Treatments were provided for five days, followed by two days of standard housing, and then five days of the other treatment. Midway through the 5-day exposure the “enrichment side” was swapped for each tank. Thus, every tank received each enrichment fully balanced for the number of zebrafish, the order of enrichment presentation, and whether the enrichment was first presented at the front or back of the tank.

A blinded observer analyzed the videos recorded during the 14-hour light period by counting the number of zebrafish on either side of the tank once every 5 minutes to measure for preference. These numbers were then subdivided for the four phases of exposure (*i*.*e*., enrichment type x enrichment side), and the preference for the enriched side figured as the total number of times zebrafish were observed on the enriched side divided by the total number of times zebrafish were observed on the barren side of the tank. This calculation avoids the bounding inherent to proportions (which vary between 0 and 1) and the associated floor and ceiling effects. However, it is asymmetric (*i*.*e*., a ratio of 1/10 *versus* 10/1 are not equally spaced from a ratio of 1/1). To correct for this effect, the ratio was logged (corresponding to the example above, in base 10, the logged values would be -1 *versus* 1, equally spaced from 0).

### Experiment 2: Tank water cortisol

Following preference testing, zebrafish were housed for a week in standard conditions (*i*.*e*., housed in barren tanks, in the same single or group housed configurations as above). For Experiment 2, alternating tanks received pebble enrichment or standard housing (barren) underneath the entirety of the tank floor. All experimental tanks could see their neighbor tanks. Visual access to conspecifics was not a treatment condition in Experiment 2. Treatments were equally distributed between singly and group housed tanks. At the end of the 7 days, we measured tank water cortisol levels, following methods described in [18]. Briefly, at the end of the experiment, we turned the tank water flow off for one hour to allow for cortisol accumulation, then collected 1mL tank water samples and froze them at -80°C. The samples were later thawed, filtered to remove biological particulates with a 33 mm, 0.45 μm, MCE syringe filter (Fisher Scientific, 09-720-005), and run through a cortisol ELISA (Cayman Chemical’s Cortisol ELISA Kit 500360) that measures free cortisol. We ran undiluted (1:1) 50 μL filtered water samples in duplicate, and we read the plate using a Varioskan LUX multimode microplate reader (wavelength 405 nm).

### Statistical analysis

Data were analyzed using General Linear Models in JMP 16 Pro, with further post-hoc tests performed in equivalent analyses using SAS 9.4 for Windows.

For the preference data (Experiment 1) each tank was observed under a 2x2 factorial of enrichment type (pebble picture *versus* conspecific visual access) crossed with enriched side (front *versus* back of the tank). Each tank was also either single housed or group housed (“number of fish”). We therefore analyzed the data as a split plot design with 2-within plot factors. Thus, Tank was nested within number of fish, and number of fish, enriched side, and enrichment type were included as main effects and second-order interactions. Tank was treated as a fixed effect as it cannot meet the criteria for a random effect (not least because 1-fish and 4-fish tanks are not equivalent units of indivisible variance drawn from the same population), following [24]. Blocking by Tank controls for other confounds such as rack and location on the rack. In a split-plot design the experimental unit is the within-plot by between-plot treatment combination.

For tank cortisol (Experiment 2), there is a single measure per tank. Thus, the analysis was controlled (blocked) by rack and location of tank on rack. The model included group *versus* singly housed and enrichment type (pebble picture *versus* barren) as crossed experimental factors, to test for both additive and interactive effects. The experimental unit is the tank.

For both analyses, the assumptions of linear models (homogeneity of variance, normality of error, and linearity) were confirmed following [25], and no transformations were required. We performed post-hoc tests using Bonferroni-corrected planned contrasts, and custom estimates, where needed. Post-hoc power was calculated as the Least Significant Number, following best practice [20].

## Results

### Experiment 1: Zebrafish preference testing

**Fig 1:**
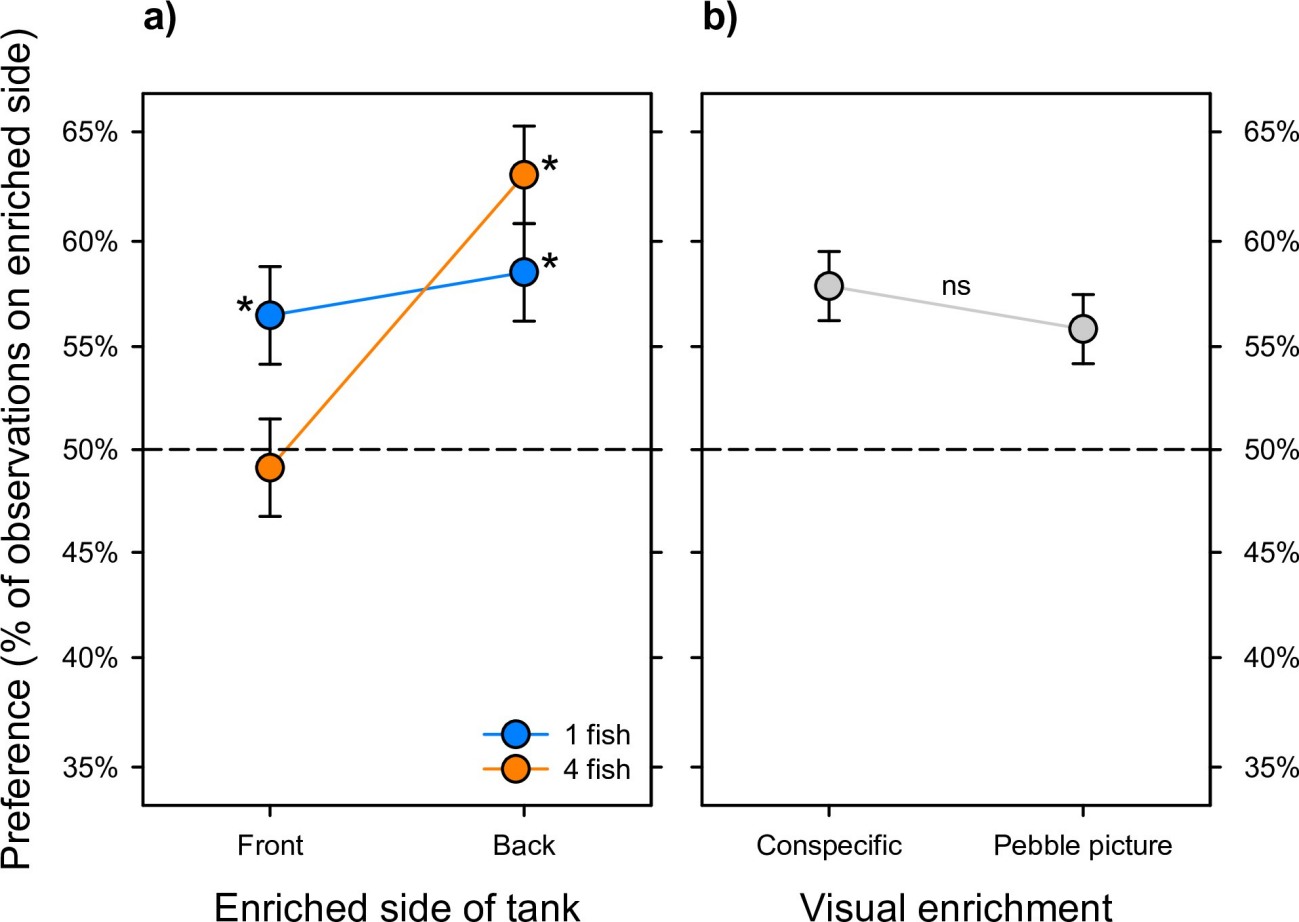
Zebrafish preference for enrichment depended on enrichment position and social housing. **a)** Singly housed zebrafish preferred enrichment regardless of position (T_1,43_ = 4.51; P<0.0001); group housed zebrafish only preferred enrichment in the back of the tank (F_1,43_ = 17.98, P = 0.0001). **b)** Preference did not differ between visual enrichment type (pebble picture *versus* visual access to conspecifics) (F_1,43_ = 0.7686; P = 0.3855). Data depict singly and group-housed fish and are plotted as LSM ± SE. * indicates LSMs with a significant preference versus the null hypothesis of no preference (dashed line).

Preference for enrichment did not differ between visual access to conspecifics and visual access to pebbles (F_1,43_ = 0.7686; P = 0.3855). A Least Significant Number power test for this difference was 523 tanks (*i*.*e*., so many tanks would be needed for this small difference to be significant that it’s not biologically relevant) [20]. Preference for both types of visual enrichments (pebble picture and visual access to conspecifics) depended on the interaction of single housing with position in the tank (F_1,43_ = 6.537, P = 0.0142), such that group housed zebrafish only preferred the enriched side of the tank when either enrichment (pebble picture or visual access) was presented in the back of the tank (F_1,43_ = 17.98, P = 0.0001); whereas singly housed zebrafish always preferred the enriched side of the tank (T_1,43_ = 4.51; P<0.0001), and did not differ in preference when the enrichment was presented at the front *versus* the back of the tank (F_1,43_ = 0.3892, P = 0.5360) (Fig 1).

### Experiment 2: Tank water cortisol

**Fig 2:**
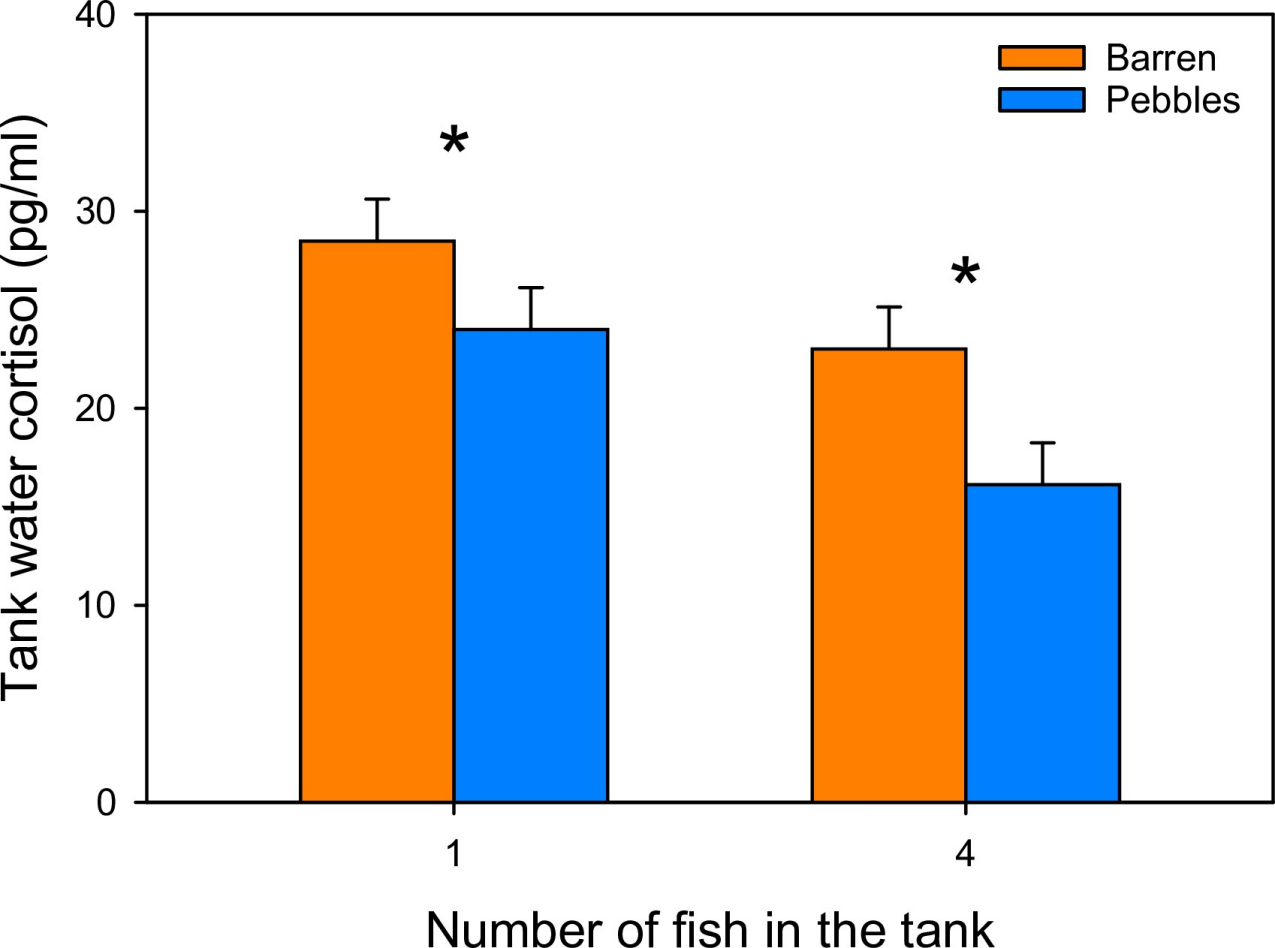
Chronic stress, measured by tank water cortisol, is reduced by social housing and pebble picture enrichment. Both enrichment with pebble pictures (F_1,10_ = 7.171; P = 0.0232) and group housing (F_1,10_ = 9.8339; P = 0.0106) significantly reduced tank water cortisol. These effects were additive and equivalent, such that singly housed enriched tanks did not differ significantly from the barren, group housed tanks (F_1,10_ = 0.1049; P = 0.7527), and group housed zebrafish with pebble pictures had the lowest cortisol levels of all treatment groups. Data are plotted as LSM ± SE. * indicates significant differences between Barren *versus* Pebble enriched tanks.

Pebble picture enriched (F_1,10_ = 7.171; P = 0.0232) and group housed (F_1,10_ = 9.8339; P = 0.0106) tanks had significantly reduced tank water cortisol levels, with group housed pebble enriched zebrafish having the lowest cortisol levels of all groups (Fig 2). The enriched-by-group housed interaction was not significant (F_1,10_ = 0.3178; P = 0.5853), suggesting that these effects were additive (Least Significant Number power test = 196 tanks). Singly housed enriched tanks did not differ significantly from the barren group housed zebrafish (F_1,10_ = 0.1049; P = 0.7527; Least Significant Number power test for this difference = 588 tanks).

## Discussion

In Experiment 1, we tested preferences for pebble pictures and visual access to neighboring conspecifics when provided in either the front or the back of the tank. Zebrafish preferred the half of the tank that was enriched, and pebble enrichment was equivalent to the positive control of visual access to conspecifics. The preference for enrichment in singly housed zebrafish was stronger than the preference for the back of the tank shown by group housed zebrafish. In Experiment 2, fish were housed in barren tanks or with pebble pictures under their tank for one week. The water cortisol analysis showed that zebrafish had significantly reduced tank water cortisol levels when housed with a pebble picture covering the bottom of the tank, with the lowest cortisol levels exhibited in group-housed zebrafish with under-tank pebble pictures. Together, these results support our hypothesis that under-tank enrichment is beneficial in real-world laboratory conditions, as shown by both zebrafish preference and physiological reactions. Experiment 2 additionally showed that the benefit of pebble enrichment was equivalent to the positive control of the group housing, which means that the inclusion of pebble picture enrichment can compensate for the stress induced by single housing. However, we stress that group housed zebrafish further benefit from pebble picture enrichment.

In Experiment 1, the two visual enrichments (conspecifics or pebble pictures) were similarly preferred. When zebrafish were singly housed, they demonstrated a preference for visual enrichment regardless of its location in the tank. Group housed zebrafish preferred the enrichment when it was in the back of the tank. We had predicted that there would be greater preference for visual access to conspecifics by singly housed zebrafish. This was true to a certain extent, as the group housed zebrafish only preferred the side of the tank with visual enrichment when it was in the back of the tank–regardless of enrichment type (pebbles or visual access to conspecifics). Given that they are already socially enriched, group housed zebrafish may have prioritized their preferred half of the tank over the addition of external environmental enrichment. We speculate that this preference for the back of the tank may be due to finding the back safer, as it is away from the occasional presence of large potential predators (*i*.*e*., humans). This result contrasts with prior findings in which there was a significant preference for the front of the tank by singly housed zebrafish [13], and thus warrants further investigation into the preference differences between singly and group housed zebrafish. Given the known preference for social interaction in zebrafish, visual access to conspecifics worked to contextualize the importance of a pebble picture to singly housed zebrafish, where the pebble picture was just as preferred as visual access to other zebrafish. Given this preference and the additive effect of social housing and pebble pictures on stress levels seen in Experiment 2, combined provision of access to conspecifics and pebble pictures below the tank is likely to have better welfare outcomes than provision of one or the other.

In Experiment 2, cortisol levels were reduced in both single and group housed zebrafish when pebble pictures were provided (in Experiment 2, visual access to conspecifics was not included as a treatment). Measuring cortisol levels in tank water is a widely used, well-validated, non-invasive method for assessing stress in zebrafish [16], and many other fish species [17]. As such, our tank water analysis showed that zebrafish enriched by a pebble picture and/or group housing experienced significantly less stress overall. The zebrafish in the least stressed state were those that had both forms of environmental enrichment provided, due to the additive effect of social housing in addition to an under-tank pebble picture. This suggests that increasing the environmental complexity and adding additional forms of biologically relevant enrichment, as opposed to only providing a single type of enrichment (*i*.*e*., social housing), promotes the overall wellbeing of zebrafish. Additionally, brine shrimp (which may be considered a form of nutritional enrichment) were provided to all zebrafish in our study. This may have improved the overall welfare of our zebrafish. The visual stimuli were still measurably beneficial, indicating that providing live food as an enrichment intervention is not enough to mitigate the effects of barren tanks on zebrafish.

Overall, much of the zebrafish literature suggests that group housed zebrafish show decreased cortisol in comparison to singly housed zebrafish, as would be expected due to zebrafish being a social species. However, this literature contains inconsistent results with regards to cortisol levels [6]. One potential explanation for this inconsistency is methodological. Commonly used ELISA kits measure total (bound + free) cortisol levels, while some kits only measure free cortisol. This distinction is critical. Thus, similar to cortisol in humans, the vast majority of the cortisol in a fish’s body is bound and not signaling [26]. Only unbound, or free, cortisol is relevant in analyzing stress levels, as it is biologically active while the bound cortisol is not [27]. The ELISA kit we used to analyze the water cortisol levels measured only free, and therefore biologically relevant, cortisol. The fact that some earlier work is unclear as to which form of cortisol is measured, emphasizes the need for both appropriate methodology and clear reporting in this literature.

### Limitations

Although the substrate photos we used were all varying portions of a single, larger natural-colored pebble picture, we still only looked at one type of substrate, which reduces the generalizability of our results. However, in conjunction with previous papers looking at preference for substrates, our experiment adds to the body of evidence that zebrafish benefit from the complexity that substrates add to their tank environments. The second limitation is that our zebrafish only received each type of treatment for five days in Experiment 1 and seven days in Experiment 2. We suggest that pebble pictures would likely be beneficial beyond the length of time we tested them for, with low risk associated with widespread use given that it is low-cost (both financially and in terms of labor) with no evidence of potential harms to the animals. It is also possible that the positive effects found could potentially diminish over a longer period of time, but further research would be needed to determine this. The third possible limitation is that our study used surplus, naive, wildtype zebrafish otherwise destined for euthanasia, and were therefore random ages and sexes. While this has the benefit of reducing effect size and producing a more generalizable result, our results are limited to adult zebrafish. In rodent research, it is understood that providing enriched environments to younger animals has lifelong benefits [28]. While we do not know how younger zebrafish may react to an under-tank pebble picture, there is a chance it would be even more beneficial for young zebrafish than it is for adult zebrafish. It has also been suggested that genetically modified zebrafish may have different reactions to environmental enrichment than wildtypes [29], but more research is needed to investigate these differences.

The intervention tested in our study is ultimately limited by the qualities of high-density laboratory aquatic housing. While we have shown that the well-being of zebrafish is improved by providing pebble pictures in addition to group housing, we do not assume that this is the best that can be done. This simple, low-cost refinement to zebrafish housing is one step towards providing zebrafish with environments that will allow them to truly thrive. Given that zebrafish are curious [30], social [6, 15], and come from much more complex natural environments [5], more complex species-appropriate housing would likely have even greater welfare benefits than what has been shown by our study.

### Future directions

Future work should establish the generalizability of these results by comparing the physiological and behavioral reactions of zebrafish to a wider variety of under-tank substrate pictures to determine if particular substrates are more preferred. Additionally, many animals benefit from novelty in enrichment, and the *Guide for the Care and Use of Laboratory Animals* states that enrichment rotation should be considered, unless the changing environment may have a negative impact on welfare [9]. Some work has already been done on neophilia in zebrafish, showing that zebrafish are curious and seek explorative opportunities [30]. Further studies should examine whether under-tank enrichment should be rotated on a schedule in order to maintain or increase the benefits of the enrichment through novelty. Potential differences in behavioral and physiological responses to enrichment between different genetic backgrounds are additionally worthy of study. While zebrafish are the most common aquatic animal model, there are a multitude of aquatic species kept in both laboratory and aquaculture settings that might benefit from under-tank enrichment. Other measures of welfare, such as breeding performance, are also worthy of study. Indeed, fecundity has been shown to increase with in-tank enrichments [31]. Therefore testing for benefits of under-tank enrichment for breeding, and other stages of the lifecycle, is warranted.

## Conclusion

When given the choice, zebrafish preferred spending their time housed over a pebble picture; this preference was equivalent to preference shown for visual access to conspecifics. Preferences for enrichment were evident whether zebrafish were housed singly or in a group, although the preference of group housed zebrafish was dependent on providing the enrichment in the back of the tank. Furthermore, pebble pictures below the tank reduced the stress levels of single and group housed zebrafish in laboratory tanks. Group housed zebrafish housed over pebble pictures had the lowest cortisol levels. The *Guide* establishes an expectation that enrichment should be provided if it is shown to improve the health and well-being of a given species, provided it doesn’t have negative impacts [9]. As under-tank enrichment has no impact on hygiene and therefore cannot disrupt the health of the zebrafish, there is no reason that under-tank enrichment should not be incorporated into zebrafish husbandry.

## Supporting information

S1 File Supporting information for Experiments 1 and 2 experimental design and schedules, and SAS code for analysis. Tables depict the application of different treatments over time in each experiment. SAS code used for data analysis is commented as needed, and can be used for replication of the analysis reported in the paper.
(PDF)
