# Supplementary material for: Pebble to the Metal: A Boulder Approach to Enrichment for Danio rerio
Source: PLoS One. 2024 May 7;19(5):e0298657. doi: 10.1371/journal.pone.0298657 (PMC11075867; doi:10.1371/journal.pone.0298657)
Supplement: S1 File — Tables depict the application of different treatments over time in each experiment. SAS code used for data analysis is commented as needed, and can be used for replication of the analysis reported in the paper. (PDF) [file pone.0298657.s001.pdf]

Supporting Information

Table S1. Experimental design and schedule for Experiment 1. The experiment ran for a total of two weeks. In Experiment 1, there were two treatments: visual access to conspecifics ("visual") and pebble pictures under the tank ("pebbles") provided in one half of the tank. F signifies that enrichment was provided at the front half of the tank; B signifies that enrichment was provided at the back half of the tank. Front and back positions of each treatment were switched on Wednesdays. Treatments are highlighted with different colors. No treatments were applied during the weekend and all fish had visual access to neighboring conspecifics at this time.

Rack	Tank	# Fish	Mon	Tues	Wed AM	Wed PM	Thu	Fri	Sat/Sun	Mon	Tues	Wed AM	Wed PM	Thu	Fri
1	1	4	Pebbles F	Pebbles F	Pebbles F	Pebbles B	Pebbles B	Pebbles B	No treatment	Visual B	Visual B	Visual B	Visual F	Visual F	Visual F
1	2	4	Pebbles B	Pebbles B	Pebbles B	Pebbles F	Pebbles F	Pebbles F	No treatment	Visual B	Visual B	Visual B	Visual F	Visual F	Visual F
1	3	1	Pebbles F	Pebbles F	Pebbles F	Pebbles B	Pebbles B	Pebbles B	No treatment	Visual B	Visual B	Visual B	Visual F	Visual F	Visual F
1	4	1	Pebbles B	Pebbles B	Pebbles B	Pebbles F	Pebbles F	Pebbles F	No treatment	Visual B	Visual B	Visual B	Visual F	Visual F	Visual F
1	5	4	Pebbles F	Pebbles F	Pebbles F	Pebbles B	Pebbles B	Pebbles B	No treatment	Visual F	Visual F	Visual F	Visual B	Visual B	Visual B
1	6	4	Pebbles B	Pebbles B	Pebbles B	Pebbles F	Pebbles F	Pebbles F	No treatment	Visual F	Visual F	Visual F	Visual B	Visual B	Visual B
1	7	1	Pebbles F	Pebbles F	Pebbles F	Pebbles B	Pebbles B	Pebbles B	No treatment	Visual F	Visual F	Visual F	Visual B	Visual B	Visual B
1	8	1	Pebbles B	Pebbles B	Pebbles B	Pebbles F	Pebbles F	Pebbles F	No treatment	Visual F	Visual F	Visual F	Visual B	Visual B	Visual B
2	9	1	Visual B	Visual B	Visual B	Visual F	Visual F	Visual F	No treatment	Pebbles F	Pebbles F	Pebbles F	Pebbles B	Pebbles B	Pebbles B
2	10	1	Visual B	Visual B	Visual B	Visual F	Visual F	Visual F	No treatment	Pebbles B	Pebbles B	Pebbles B	Pebbles F	Pebbles F	Pebbles F
2	11	4	Visual B	Visual B	Visual B	Visual F	Visual F	Visual F	No treatment	Pebbles F	Pebbles F	Pebbles F	Pebbles B	Pebbles B	Pebbles B
2	12	4	Visual B	Visual B	Visual B	Visual F	Visual F	Visual F	No treatment	Pebbles B	Pebbles B	Pebbles B	Pebbles F	Pebbles F	Pebbles F
2	13	1	Visual F	Visual F	Visual F	Visual B	Visual B	Visual B	No treatment	Pebbles F	Pebbles F	Pebbles F	Pebbles B	Pebbles B	Pebbles B
2	14	1	Visual F	Visual F	Visual F	Visual B	Visual B	Visual B	No treatment	Pebbles B	Pebbles B	Pebbles B	Pebbles F	Pebbles F	Pebbles F
2	15	4	Visual F	Visual F	Visual F	Visual B	Visual B	Visual B	No treatment	Pebbles F	Pebbles F	Pebbles F	Pebbles B	Pebbles B	Pebbles B
2	16	4	Visual F	Visual F	Visual F	Visual B	Visual B	Visual B	No treatment	Pebbles B	Pebbles B	Pebbles B	Pebbles F	Pebbles F	Pebbles F

Table S2. Experimental design and schedule for Experiment 2. The experiment ran for one week. Treatments (barren tanks or tanks with pebbles under the entirety of the tank bottom) are highlighted with different colors. All fish had visual access to conspecifics during this experiment, and only one treatment was provided to each tank.

Rack	Tank	# Fish	Mon	Tues	Wed	Thu	Fri	Sat	Sun	Mon
1	1	4	Barren	Barren	Barren	Barren	Barren	Barren	Barren	Water sample taken
1	2	4	Pebbles	Pebbles	Pebbles	Pebbles	Pebbles	Pebbles	Pebbles	Water sample taken
1	3	1	Barren	Barren	Barren	Barren	Barren	Barren	Barren	Water sample taken
1	4	1	Pebbles	Pebbles	Pebbles	Pebbles	Pebbles	Pebbles	Pebbles	Water sample taken
1	5	4	Barren	Barren	Barren	Barren	Barren	Barren	Barren	Water sample taken
1	6	4	Pebbles	Pebbles	Pebbles	Pebbles	Pebbles	Pebbles	Pebbles	Water sample taken
1	7	1	Barren	Barren	Barren	Barren	Barren	Barren	Barren	Water sample taken
1	8	1	Pebbles	Pebbles	Pebbles	Pebbles	Pebbles	Pebbles	Pebbles	Water sample taken
2	9	1	Barren	Barren	Barren	Barren	Barren	Barren	Barren	Water sample taken
2	10	1	Pebbles	Pebbles	Pebbles	Pebbles	Pebbles	Pebbles	Pebbles	Water sample taken
2	11	4	Barren	Barren	Barren	Barren	Barren	Barren	Barren	Water sample taken
2	12	4	Pebbles	Pebbles	Pebbles	Pebbles	Pebbles	Pebbles	Pebbles	Water sample taken
2	13	1	Barren	Barren	Barren	Barren	Barren	Barren	Barren	Water sample taken
2	14	1	Pebbles	Pebbles	Pebbles	Pebbles	Pebbles	Pebbles	Pebbles	Water sample taken
2	15	4	Barren	Barren	Barren	Barren	Barren	Barren	Barren	Water sample taken
2	16	4	Pebbles	Pebbles	Pebbles	Pebbles	Pebbles	Pebbles	Pebbles	Water sample taken

SAS code for original data and analysis.

- The following code can be cut and pasted into SAS and run to replicate the analyses reported in the paper.
- The code is commented as needed.

Preference testing

```
DATA ZebraFishPrefData; INPUT  Tank NumFish Treatment &$16. EnrichedSide &$16. Log2EoverB; Lines;
1  4  Gravel  F  0.3016557
1  4  Gravel  B  0.866733469
1  4  Blinder  B  0.610053482
1  4  Blinder  F  0.423807709
2  4  Gravel  B  0.855795277
2  4  Gravel  F  0.291530742
2  4  Blinder  B  0.704993204
2  4  Blinder  F  -0.434049468
3  1  Gravel  F  -0.444302094
3  1  Gravel  B  0.385653692
3  1  Blinder  B  0.955145764
3  1  Blinder  F  0.56828376
4  1  Gravel  B  0.910732662
4  1  Gravel  F  0.509674373
4  1  Blinder  B  0.321928095
4  1  Blinder  F  1.231946728
5  4  Gravel  F  0.362570079
5  4  Gravel  B  0.910732662
5  4  Blinder  F  0.57870351
5  4  Blinder  B  1.518923739
6  4  Gravel  B  0.030057234
6  4  Gravel  F  -0.91753784
6  4  Blinder  F  0.516398214
6  4  Blinder  B  1.091147888
7  1  Gravel  F  0.403355694
7  1  Gravel  B  0.344648171
7  1  Blinder  F  0.610053482
7  1  Blinder  B  0.403355694
8  1  Gravel  B  0.160464672
8  1  Gravel  F  0.468148836
8  1  Blinder  F  0.251538767
8  1  Blinder  B  0.444302094
9  1  Blinder  B  0.20069735
9  1  Blinder  F  0.2410081
9  1  Gravel  F  1.045323991
9  1  Gravel  B  -0.851136614
10  1  Blinder  B  1.693896872
10  1  Blinder  F  -0.485426827
10  1  Gravel  B  1.184424571
10  1  Gravel  F  -0.321928095
11  4  Blinder  B  0.426814667
11  4  Blinder  F  -0.888682977
11  4  Gravel  F  0.631032149
11  4  Gravel  B  0.56828376
12  4  Blinder  B  0.362570079
12  4  Blinder  F  -1.056731885
12  4  Gravel  B  1.045323991
12  4  Gravel  F  -0.120294234
13  1  Blinder  F  1
13  1  Blinder  B  0.694373717
13  1  Gravel  F  0.910732662
13  1  Gravel  B  0.019899557
14  1  Blinder  F  0.120294234
14  1  Blinder  B  0.694373717
14  1  Gravel  B  0.403355694
14  1  Gravel  F  -0.080170349
15  4  Blinder  F  -0.33207605
15  4  Blinder  B  1.255944981
15  4  Gravel  F  0.020037753
```

```
15  4  Gravel  B  0.589138177
16  4  Blinder  F  -0.56828376
16  4  Blinder  B  1.532013621
16  4  Gravel  B  -0.020037753
16  4  Gravel  F  0.372752354
;
RUN;

/* Here we use PROC MIXED rather than PROC GLM in order to take advantage of the
LSMESTIMATE command for post-hoc tests. The analysis is otherwise equivalent to a
LS GLM, and gives the same results*/

PROC MIXED  DATA=ZebraFishPrefData ALPHA=0.0125;
CLASS Tank NumFish Treatment EnrichedSide;
MODEL  Log2EoverB =  Tank(NumFish ) NumFish Treatment EnrichedSide NumFish*Treatment
NumFish*EnrichedSide Treatment*EnrichedSide;

/*The LSMEANS command generates LSM and SE for graphing. This also generates p values to
ask if individual means differ from zero (no preference in log space). These p values
have a critical alpha of 0.05/4=0.0125 to correct for multiple comparisons.
The SLICE option tests whether the fish differ in preference for the
the front or the back of the tank, split out by single housed, and group-housed.
These p-values have a critical alpha of 0.05/2=0.025 to correct for multiple comparisons*/
LSMEANS NumFish*EnrichedSide/SLICE=numfish;

/*The LSMESTIMATE command generates an ESTIMATE equivalent matrix to test whether
the mean preference for singly housed fish is significantly greater than 0
(no preference in log space)*/
LSMESTIMATE    NumFish*EnrichedSide "mean single preference" 0.5 0.5 0 0 ;

RUN;
```

Tank cortisol

```
/* Here we use PROC MIXED rather than PROC GLM simply because
PROC MIXED gives less redundant and more detailed output by default.
As before, this is equivalent to a LS GLM*/

DATA TankCortisol; INPUT  WaterCortisol Treatment &$16. NumFish Location &$16. Rack; Lines;
22  Barren  4  Edge  0
17.7  Gravel  4  Middle  0
36.4  Barren  1  Middle  0
25.6  Gravel  1  Edge  0
17.6  Barren  4  Edge  0
11  Gravel  4  Middle  0
24.7  Barren  1  Middle  0
21  Gravel  1  Edge  0
25.6  Barren  1  Edge  1
23.7  Gravel  1  Middle  1
23.3  Barren  4  Middle  1
19.8  Gravel  4  Edge  1
27.3  Barren  1  Edge  1
25.7  Gravel  1  Middle  1
29.2  Barren  4  Middle  1
16  Gravel  4  Edge  1
;
RUN;

PROC MIXED DATA=TankCortisol ALPHA=0.05;
CLASS Treatment NumFish Location Rack;
MODEL  WaterCortisol =  Location Rack Treatment NumFish Treatment*NumFish;
LSMEANS Treatment*NumFish;
RUN;
```
